# A Case of Hare-lip

**Published:** 1880-04

**Authors:** George W. Orr

**Affiliations:** Physician and Surgeon


### A CASE OF HARE-LIP.

Willie F., aged nine, together with his father, called at my office in November, 1879. The little fellow had an ugly looking lip and nose, the lip was divided a little to the left of the median line and the division extended into the nostril. Both hard and soft palates were cleft and the two halves of the jaw were more than a quarter of an inch apart, and the front teeth were quite prominent, the left wing of the nose was drawn down and out. I promised Mr. F., that I could improve his son's appearance, and would operate as soon as I could get a physician to administer chloroform. I wrote Dr. Lawbaugh a line, and he was kind enough to drive five miles in a bad storm. On December 9, after the little fellow was thoroughly under the influence of the anæsthetic, I divided freely the connective tissue between the lip and gum on both sides, freshened the gums on the right side and took quite a liberal strip from each side of the fissure, drew the wing of the nose up to its proper place hoping it would unite with the freshened part of the opposite gum. I placed strong silver pins through the lip, the upper as near the junction of lip and nose as possible. I strapped the face firmly. No bad effects from the chloroform followed. On the third day I removed the pins. Everything appeared firm. On the sixth day I removed the straps. You can not imagine with what pride and satisfaction I viewed the result of my first hare-lip operation. With more than satisfaction, I pointed to the complete union and accuracy of the junction of the mucous membrane, and the wing of the nose was as perfect as its fellow. I immediately restrapped the face, and assured the mother it was a first-class result. But later on, I found I had been too sanguine. Nearly a week after, I removed the straps and found the wing of the nose had slid down and out a trifle, and had drawn the left half of the lip up a little leaving a little notch in the lower margin of the lip. The result is as good if not better than I promised, the two halves of the superior maxillary are drawn together over an eighth of an inch since the operation.

GEORGE W. ORR, M. D.
*Physician and Surgeon.*

